# Associations of Bubble Tea Consumption with Sleep Disturbance and Anxiety in Adolescents: Findings from the Zhejiang Childhood Behavior and Health Cohort

**DOI:** 10.3390/nu18121960

**Published:** 2026-06-17

**Authors:** Xiangyu Chen, Mingbin Liang, Lijin Chen, Weiyuan Yao, Qingfang He, Min Yu, Meng Wang

**Affiliations:** Department of Non-Communicable Disease Control and Prevention, Zhejiang Provincial Center for Disease Control and Prevention, Hangzhou 310051, China; xychen@cdc.zj.cn (X.C.); mbliang@cdc.zj.cn (M.L.); ljch@cdc.zj.cn (L.C.); ywyao@cdc.zj.cn (W.Y.); qfhe@cdc.zj.cn (Q.H.); myu@cdc.zj.cn (M.Y.)

**Keywords:** adolescents, sleep disturbance, bubble tea consumption, anxiety, mediation analysis

## Abstract

**Objectives**: We aimed to examine the association between bubble tea consumption and anxiety symptoms among adolescents in Eastern China and to explore the potential role of sleep disturbance in the observed association between bubble tea consumption and anxiety symptoms. **Methods**: This study utilized cross-sectional baseline data from the Zhejiang Childhood Behavior and Health Cohort. Bubble tea consumption frequency was categorized as 0, 1–2, and ≥3 days per week. Anxiety symptoms were assessed using the Generalized Anxiety Disorder—7 (GAD-7) scale, while sleep disturbance was measured through self-reported items. Associations between bubble tea consumption and anxiety symptoms were examined using multivariable logistic regression models, and dose–response relationships were evaluated with restricted cubic spline (RCS) models. Subgroup analyses stratified by age, sex, school type, residence, and body mass index (BMI) were conducted to assess the consistency of the associations. An exploratory mediation analysis with bootstrap confidence intervals was performed to evaluate the indirect association through sleep disturbance. Sensitivity analyses using a stricter definition of anxiety symptoms (GAD-7 ≥ 10) were conducted to assess robustness. **Results**: A total of 11,847 adolescents aged 12–18 years were included, of whom 32.03% met the GAD-7 threshold for any anxiety symptoms (GAD-7 ≥ 5, including mild symptoms). Compared with non-consumers, adolescents consuming bubble tea 1–2 days per week had higher odds of anxiety (OR = 1.12, 95% CI: 1.02–1.22), while those consuming bubble tea ≥3 days per week had substantially higher odds (OR = 1.53, 95% CI: 1.30–1.80). Each additional day of bubble tea consumption per week was associated with 10% higher odds of anxiety (OR = 1.10, 95% CI: 1.06–1.14). RCS analysis demonstrated a significant positive linear association between bubble tea consumption and anxiety (*p* for non-linearity > 0.05). Associations were consistent across age, sex, school type, residence, and BMI categories (all *p* for interaction > 0.05). Sensitivity analyses yielded similar results. Exploratory mediation analysis suggested that sleep disturbance may be statistically related to a portion of the observed association between bubble tea consumption and anxiety symptoms. **Conclusions**: Higher frequency of bubble tea consumption was associated with greater odds of anxiety symptoms among adolescents in a dose–response pattern. Sleep disturbance may statistically explain part of the association. These findings should be considered hypothesis-generating and require confirmation in prospective longitudinal studies.

## 1. Introduction

Adolescent mental health has become a major public health concern worldwide [1,2]. Anxiety disorders are among the leading contributors to non-fatal disease burden during adolescence [3]. Pooled estimates from the Global Burden of Disease Study indicate that anxiety disorders account for a substantial proportion of years lived with disability among individuals aged 10–19 years [4]. Depending on the region and assessment method, prevalence estimates vary between 5% and 10%, while research indicates that by age 18, the average lifetime prevalence of any anxiety disorder across childhood and adolescence is around 15–20% [5,6]. Previous studies have reported a clear increase in anxiety prevalence over time. For example, a large school-based survey found that the proportion of adolescents meeting anxiety screening criteria increased from 34.1% in 2012 to 44.0% in 2018 [7]. Consistent with these global trends, regional evidence from China also indicates a high burden of anxiety symptoms; a study conducted in Zhejiang Province reported a prevalence of 31.3% among adolescents [8]. This pattern appears more pronounced among girls and adolescents from lower socioeconomic backgrounds [9]. Adolescence is characterized by rapid neurodevelopment and hormonal changes, along with increasing autonomy in health-related behaviors [10]. These features make it a sensitive period during which environmental exposures may influence mental health trajectories.

Parallel to these trends, the consumption of sugar-sweetened and caffeinated beverages has increased among adolescents, especially in China, where bubble tea is widely consumed [11,12,13]. Bubble tea, also referred to as milk tea, is a tea-based beverage prepared with tea, milk, and additional ingredients [14]. Population-based surveys in China suggest that a substantial proportion of adolescents consume bubble tea at least once per week [14], with higher intake observed in urban areas and among older students [15]. Bubble tea typically contains high levels of added sugars and, in many formulations, caffeine derived from tea, both of which have been associated with neurobehavioral outcomes. However, evidence regarding the association between bubble tea consumption and mental health outcomes, particularly anxiety, remains inconsistent. Some studies have reported that bubble tea consumption is associated with increased risk of anxiety symptoms [11,13], whereas one longitudinal study of 686 male college students reported that the association was no longer statistically significant after adjustment for potential confounders, including computer usage time [16]. These inconsistencies may reflect differences in study design, population characteristics, and confounder control. Therefore, more large-sample studies are needed to examine this relationship, particularly in adolescent populations.

Sleep disturbance has been proposed as a potential factor associated with both dietary behaviors and mental health outcomes in adolescents [17]. Experimental and observational studies have shown that caffeine intake can delay sleep onset, reduce sleep duration, and impair sleep quality, while high sugar intake may disrupt circadian regulation and metabolic homeostasis [18,19,20]. In turn, sleep disturbance has been consistently associated with anxiety symptoms [21]. However, most prior studies have examined these associations separately. Few studies have explored the potential role of sleep disturbance in the association between beverage consumption and anxiety. In addition, many existing studies rely on simplified exposure measures, have incomplete adjustment for confounding, or do not adequately account for adolescent-specific behavioral contexts.

These gaps highlight the need for population-based studies that integrate dietary behavior, sleep health, and mental health within a unified analytical framework. To address these limitations, the present study utilized baseline data from the Zhejiang Childhood Behavior and Health Cohort, a large school-based sample of Chinese adolescents with detailed behavioral and psychosocial measures. By focusing on bubble tea consumption as a culturally relevant exposure and employing a multi-item assessment of sleep disturbance alongside a validated measure of anxiety symptoms, this study provides a more specific and contextually relevant evaluation of these interrelationships.

In this cross-sectional study, we examined the association between bubble tea consumption and anxiety symptoms among adolescents and explored whether sleep disturbance statistically accounted for part of this association. We hypothesized that more frequent bubble tea consumption would be associated with greater odds of anxiety symptoms and that sleep disturbance would be statistically related to the observed association. The findings may help inform future longitudinal studies designed to clarify the temporal relationships among bubble tea consumption, sleep disturbance, and anxiety symptoms.

## 2. Materials and Methods

### 2.1. Study Design and Participants

**Figure 1:**
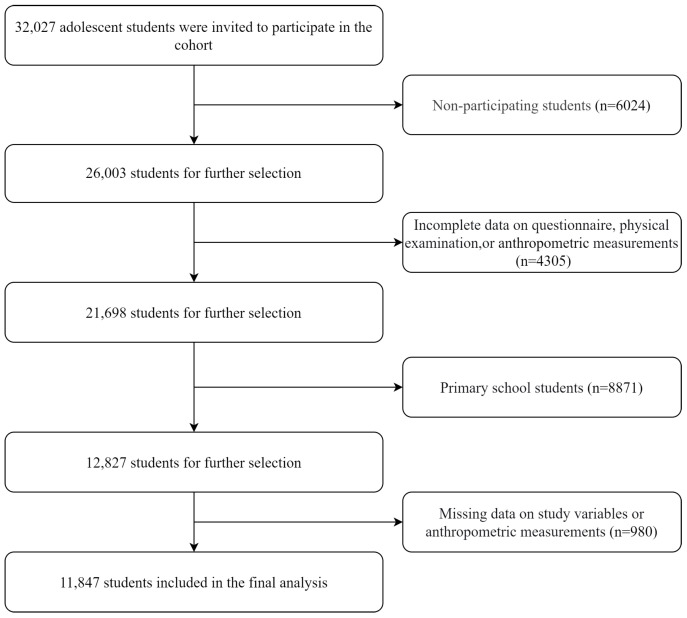
Flowchart of the participant selection process.

This study used cross-sectional baseline data from the Zhejiang Childhood Behavior and Health Cohort, a large school-based cohort established in Zhejiang Province, China, between September 2024 and January 2025. Detailed information on the cohort design has been described elsewhere [22]. For the present analysis, only middle and high school students were included because anxiety symptoms were assessed using the Generalized Anxiety Disorder 7-item scale (GAD-7), which was administered exclusively to this subgroup. Primary school students completed a different questionnaire that did not include the GAD-7. Among 26,003 participants in the baseline survey, 11,847 adolescents with complete data on exposure, mediator, outcome, and covariates were included in the primary analysis. The participant selection process is illustrated in Figure 1.

### 2.2. Data Collection

A self-administered paper-based questionnaire, adapted from the Youth Risk Behavior Surveillance System and the Global School-based Student Health Survey, was used to collect information on demographics, dietary behaviors (including bubble tea consumption frequency over the past week), sleep status, physical activity, substance use, and mental health. Anthropometric measurements, including height and weight, were obtained by trained healthcare providers. All questionnaire data were double-entered using EpiData 4.7, and unique participant codes were used for data linkage.

### 2.3. Quality Control Measures

Before the baseline survey, each study site established a research team composed of personnel with medical training and field experience. Roles and responsibilities were clearly defined. A standardized field operations manual was developed, and all team members completed uniform training to reduce systematic error. A fieldwork log was maintained to document survey progress. On-site supervision was carried out by project staff throughout data collection. At the end of each survey day, designated personnel reviewed and verified all questionnaires in a timely manner. Physical examinations, including anthropometric measurements, were conducted by trained healthcare professionals from local health centers or township hospitals, following standardized protocols issued by the education authorities.

### 2.4. Definition of Variables

Anxiety symptoms were measured using the GAD-7 [23]. The GAD-7 assesses symptom frequency over the past two weeks on a 0–3 scale, with total scores ranging from 0 to 21. A score ≥ 5 was used to identify any anxiety symptoms (including mild symptoms) in the primary analysis, in line with previous adolescent studies. This cut-off reflects a broad screening approach rather than a clinical diagnosis of generalized anxiety disorder. As a sensitivity analysis, a higher cut-off (≥10) was applied to define moderate-to-severe anxiety [24]. Bubble tea consumption was assessed using a self-administered item: “During the past 7 days, how many days did you consume milk tea?” Bubble tea was defined as beverages primarily made from milk and tea, with optional toppings (e.g., tapioca pearls, red beans, chocolate). Responses ranged from 0 to 7 days/week. In dose–response and subgroup analyses, weekly bubble tea consumption frequency was treated as a continuous variable (0–7 days/week). For regression analyses, participants were categorized as non-consumers (0 day/week), moderate consumers (1–2 days/week), and frequent consumers (3–7 days/week). For descriptive analyses, bubble tea consumption was further dichotomized as non-consumption (0 day/week) and any consumption (≥1 day/week). Sleep disturbance was assessed using five self-reported items referring to the previous 30 days: (1) snoring or breathing disturbance during sleep, (2) difficulty initiating sleep (>30 min), (3) nocturnal awakening (≥2 times per night), (4) early morning awakening, and (5) daytime sleepiness affecting academic performance. Each item was rated on a three-point scale: 1 = ≥3 days/week, 2 = 1–2 days/week, and 3 = almost never. A binary variable was created, and participants were classified as having sleep disturbance if they reported at least one sleep-related symptom occurring on three or more days per week during the previous 30 days. Covariates were selected a priori based on previous literature and included sociodemographic characteristics, lifestyle factors, body weight status, screen time, fast-food consumption, and school-related stress. Sociodemographic variables included age, sex, residential area (urban/rural), school type (middle school/high school), dormitory status (living at home/school dormitory), only-child status (yes/no), and paternal and maternal education levels (secondary school or below, senior high school, and college or above). Lifestyle factors included regular exercise, smoking, alcohol drinking, screen time, and fast-food consumption. Regular exercise was defined as engaging in at least 60 min of moderate-to-vigorous physical activity per day [25]. Smoking was defined as smoking on at least one day during the previous 30 days, and alcohol drinking was defined as consuming alcohol on at least one day during the previous 30 days. Screen time was assessed by the question: “During the past week, on average, how many hours per day did you spend on screen-based activities, including smartphones, computers, tablets, television, and video games?” Daily screen time was dichotomized into low screen time (<2 h/day) and high screen time (≥2 h/day) [26]. Fast-food consumption was assessed using the question: “During the past 7 days, on how many days did you consume fast-food (e.g., McDonald’s, KFC, or other fast-food restaurants serving hamburgers, fried chicken, and French fries)?” Responses ranged from 0 to 7 days per week and were categorized as no consumption (0 day/week) or any consumption (≥1 day/week). Body mass index (BMI) was calculated as weight in kilograms divided by height in meters squared (kg/m^2^) using measured height and weight. According to the “Overweight and Obesity Screening Table for School-age Children and Adolescents” published and implemented by the health industry standard of the People’s Republic of China [27]. BMI was classified according to sex- and age-specific cutoffs. Participants were categorized into a binary variable: normal and overweight/obesity. School-related stress was approximated using self-rated academic performance, which may reflect perceived academic pressure. Participants were asked: “During the past semester, how would you rate your academic performance compared with your classmates?” Response options included excellent, average, and below average. This variable was treated as an ordinal categorical variable, with excellent performance serving as the reference category in regression analyses.

### 2.5. Statistical Methods

Continuous variables were summarized as means and standard deviations (SDs). Categorical variables were presented as frequencies and percentages. Differences between groups were assessed using independent-samples *t*-tests for continuous variables, and chi-square tests for categorical variables. Multivariable logistic regression models were used to examine the associations between bubble tea consumption, sleep disturbance, and anxiety symptoms. Three sequential models were constructed: Model 1 was unadjusted; Model 2 adjusted for age and sex; and Model 3 further adjusted for BMI category, residence, parental education, school type, dormitory status, only-child status, smoking, alcohol drinking, regular physical activity, screen time, fast-food consumption, and self-rated academic performance. Odds ratios (ORs) and corresponding 95% confidence intervals (CIs) were reported. To examine the shape of the association between bubble tea consumption frequency and anxiety symptoms, restricted cubic spline (RCS) models were fitted with three knots placed at the 10th, 50th, and 90th percentiles of the exposure distribution. Given the limited range of exposure values (0–7 days/week), a parsimonious three-knot specification was adopted to minimize overfitting. The median exposure level (1 day/week) was used as the reference value. RCS models were adjusted for the same covariates included in Model 3. Subgroup analyses were performed to evaluate the consistency of the association between bubble tea consumption and anxiety symptoms across age groups (≤13, 14–15, and ≥16 years), sex, school type (middle school, high school), residence (urban, rural), BMI category (normal, overweight/obesity). Effect modification was assessed by including interaction terms between bubble tea consumption and each stratification variable in the fully adjusted models. A two-sided *p*-value < 0.05 for the interaction term was considered statistically significant. Several sensitivity analyses were conducted to evaluate the robustness of the findings. First, the primary analyses were repeated using a stricter definition of anxiety symptoms (GAD-7 ≥ 10) to assess whether the results were sensitive to the choice of anxiety threshold. Second, to account for the clustered nature of the school-based data, mixed-effects logistic regression models with school included as a random intercept were fitted. Multicollinearity among independent variables was assessed using variance inflation factors (VIFs), with VIF values > 5 considered indicative of substantial collinearity. An exploratory mediation analysis was conducted to statistically decompose the observed association between bubble tea consumption and anxiety symptoms and to evaluate the extent to which sleep disturbance was associated with this relationship. The analysis was performed using the mediation package in R. Direct and indirect associations were estimated using a nonparametric bootstrap procedure with 5000 resamples to obtain bias-corrected 95% confidence intervals. All statistical analyses were conducted using R software (version 4.2.2; R Foundation for Statistical Computing, Vienna, Austria). All tests were two-sided, and statistical significance was defined as *p* < 0.05.

## 3. Results

### 3.1. General Characteristics of the Participants

A total of 11,847 adolescents aged 12–18 years were included in the analysis, with a mean age of 13.66 ± 1.49 years. Table 1 summarizes participant characteristics according to anxiety status. Overall, 32.03% of participants were classified as having any anxiety symptoms (GAD-7 ≥ 5, including mild symptoms). Compared with those without anxiety symptoms, participants with anxiety symptoms were older, more likely to be female (both *p* < 0.001), and had a lower prevalence of overweight/obesity (*p* < 0.05). Significant differences were also observed in school type, accommodation type, and parental education level (all *p* < 0.001). Adolescents with anxiety symptoms reported higher frequencies of bubble tea consumption, sleep disturbance, smoking, alcohol consumption, fast-food consumption, and screen time ≥ 2 h/day (all *p* < 0.001), whereas regular physical activity was less common (*p* < 0.001). Self-rated academic performance also differed significantly between groups (*p* < 0.001). No significant differences were observed in residence or only-child status (both *p* > 0.05).

### 3.2. Prevalence of Anxiety, Bubble Tea Consumption and Sleep Disturbance by Participant Characteristics

Table 2 shows the prevalence of anxiety symptoms, bubble tea consumption (≥1 day/week), and sleep disturbance across participant characteristics. Significant differences in anxiety symptoms and sleep disturbance were observed across age groups (both *p* < 0.001), whereas bubble tea consumption did not differ by age (*p* > 0.05). Girls had higher prevalence of anxiety symptoms, bubble tea consumption, and sleep disturbance than boys (all *p* < 0.001). Bubble tea consumption was more common among urban than rural adolescents (*p* < 0.001), whereas anxiety symptoms and sleep disturbance did not differ by residence. Overweight/obese adolescents had lower prevalence of anxiety symptoms but higher prevalence of sleep disturbance (both *p* < 0.05) than normal-weight adolescents, while bubble tea consumption was similar across BMI categories (*p* > 0.05). Adolescents with high screen time, smoking, or alcohol consumption had higher prevalence of anxiety symptoms, bubble tea consumption, and sleep disturbance (all *p* < 0.001). Fast-food consumption was associated with higher prevalence of anxiety symptoms and bubble tea consumption, but not sleep disturbance. Regular physical activity was associated with lower prevalence of anxiety symptoms and sleep disturbance (both *p* < 0.001). In addition, significant differences in all three outcomes were observed across levels of self-rated academic performance (all *p* < 0.001).

### 3.3. Multivariable Logistic Regression Analysis of Factors Associated with Anxiety

Table 3 presents the results of the multivariable logistic regression models examining the associations between participant characteristics and anxiety symptoms. Older age and female sex were independently associated with higher odds of anxiety symptoms (both *p* < 0.001). Girls had significantly higher odds of reporting anxiety symptoms than boys (*p* < 0.001). Living in a school dormitory was associated with higher odds of anxiety (*p* < 0.001), whereas higher maternal education level was associated with lower odds (*p* < 0.01). Overweight/obesity was associated with modestly lower odds of anxiety symptoms compared with normal weight (*p* < 0.05). Regarding behavioral characteristics, higher frequency of bubble tea consumption was positively associated with anxiety symptoms. Sleep disturbance was strongly associated with anxiety symptoms (OR = 5.00, 95% CI: 4.57–5.48). Smoking, alcohol consumption, and fast-food consumption were also positively associated with anxiety symptoms, whereas regular physical activity was inversely associated with anxiety symptoms (all *p* < 0.01). In addition, lower self-rated academic performance was associated with higher odds of anxiety symptoms (all *p* < 0.05). No statistically significant associations were observed for school type, residence, only-child status, paternal education level, or screen time.

### 3.4. Multivariable Regression Analysis of Bubble Tea Consumption Frequency in Relation to Anxiety

Table 4 presents the associations between bubble tea consumption frequency and anxiety symptoms under three levels of adjustment. In the continuous analysis, higher bubble tea consumption frequency was consistently associated with higher odds of anxiety symptoms across all models. In the fully adjusted model (Model 3), each additional day of bubble tea consumption per week was associated with 10% higher odds of anxiety symptoms (OR = 1.10, 95% CI: 1.06–1.14). Similar findings were observed in the categorical analysis. Compared with adolescents who did not consume bubble tea, those who consumed bubble tea 1–2 days per week had higher odds of anxiety symptoms (OR = 1.12, 95% CI: 1.02–1.22), whereas those who consumed bubble tea 3–7 days per week had substantially higher odds (OR = 1.53, 95% CI: 1.30–1.80) in the fully adjusted model. The associations remained statistically significant after adjustment for potential confounding variables. Significant positive trends were observed across increasing categories of bubble tea consumption in all models (all *p* for trend < 0.001), indicating that higher consumption frequency was associated with progressively higher odds of anxiety symptoms.

### 3.5. Dose–Response Analysis of Bubble Tea Consumption and Anxiety

**Figure 2:**
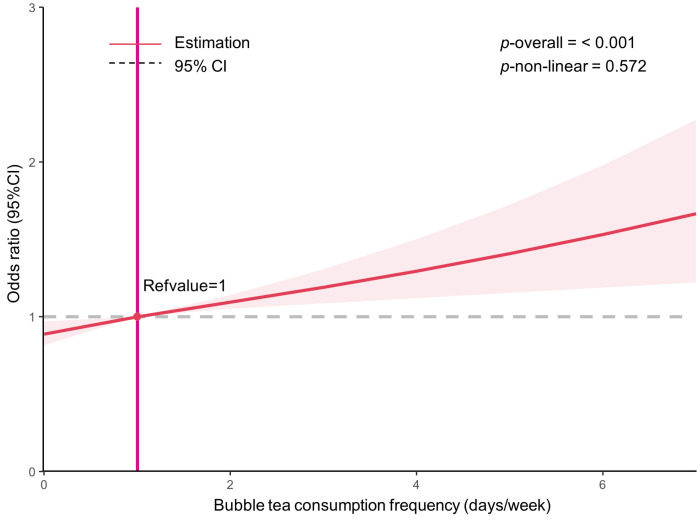
Dose–response association between weekly bubble tea consumption days and anxiety. Restricted cubic spline (RCS) model was used to estimate the association between weekly bubble tea consumption days (0–7 days/week) and the odds of anxiety, with 95% confidence intervals (CIs). The x-axis represents weekly bubble tea consumption days, and the y-axis represents the odds ratios (ORs) for anxiety. The solid line indicates the estimated association, and the shaded area represents the corresponding 95% CIs. The horizontal dashed line at OR = 1.0 indicates no association. The vertical solid line indicates the 50th percentile (median; reference value) of weekly bubble tea consumption days (1 day/week). Model was adjusted for age, sex, BMI, residence, parental education, school type, dormitory status, only-child status, smoking, drinking, fast-food consumption, self-rated academic performance, screen time, and regular exercise.

Figure 2 presents the dose–response relationship between weekly bubble tea consumption frequency and the odds of anxiety symptoms, estimated using an RCS model based on the fully adjusted logistic regression. Covariates included age, sex, BMI, residence, parental education, school type, dormitory status, only-child status, smoking, alcohol consumption, fast-food consumption, self-rated academic performance, screen time, and regular exercise. A significant positive linear association was observed between weekly bubble tea consumption and anxiety symptoms (*p* for overall association < 0.001; *p* for non-linearity > 0.05). The odds of anxiety symptoms increased progressively with higher weekly bubble tea consumption frequency, indicating a linear dose–response relationship.

### 3.6. Subgroup Analysis

**Figure 3:**
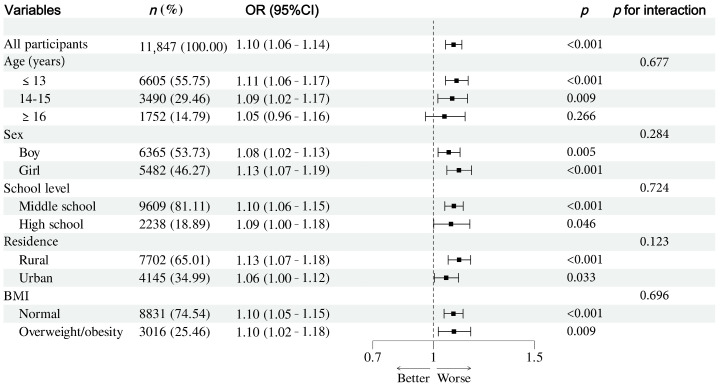
Subgroup analyses for the association between bubble tea consumption frequency and anxiety. Subgroups included age, sex, school type, residence and body mass index (BMI). Odds ratios and 95% confidence intervals were estimated from fully adjusted models, with the corresponding stratification variable excluded from each model. Models were adjusted for age, sex, BMI, residence, parental education, school type, dormitory status, only-child status, smoking, drinking, fast-food consumption, self-rated academic performance, screen time, and regular exercise. The black dots represent OR point estimates, horizontal lines indicate 95% confidence intervals, and the vertical dashed line indicates the null value (OR = 1). Abbreviations: BMI, body mass index; OR, odds ratio; CI, confidence interval.

Subgroup analyses demonstrated that the positive association between bubble tea consumption frequency and anxiety symptoms was generally consistent across the predefined subgroups examined (Figure 3). Similar associations were observed across age groups, sex, school type, residence, and BMI categories. No statistically significant interactions were identified between bubble tea consumption frequency and any subgroup variable (all *p* for interaction > 0.05), suggesting that the observed association between bubble tea consumption and anxiety symptoms was broadly consistent across these population subgroups.

### 3.7. Sensitivity Analyses

Several sensitivity analyses were conducted to evaluate the robustness of the findings. First, to account for potential clustering of students within schools, we re-estimated the primary models using mixed-effects logistic regression models with school included as a random intercept. The effect estimates remained largely unchanged (Table S1), suggesting that the observed associations were robust after accounting for school-level clustering. Given the similarity of the results and for ease of interpretation, the original logistic regression models were retained as the primary analyses. Second, analyses were repeated using a more stringent definition of anxiety (GAD-7 ≥ 10), corresponding to moderate-to-severe symptoms. The results were consistent with the primary analysis; for example, compared with non-consumers, adolescents consuming bubble tea ≥3 days per week had OR = 1.46 (95% CI: 1.16–1.83) (Table S2). RCS analyses again indicated a positive linear dose–response relationship (*p* for nonlinearity > 0.05) (Figure S1). Overall, these sensitivity analyses demonstrated that the observed association between bubble tea consumption and anxiety was robust to alternative outcome definitions and adjustment for school-level clustering.

### 3.8. Exploratory Mediation Analysis Involving Sleep Disturbance

**Figure 4:**
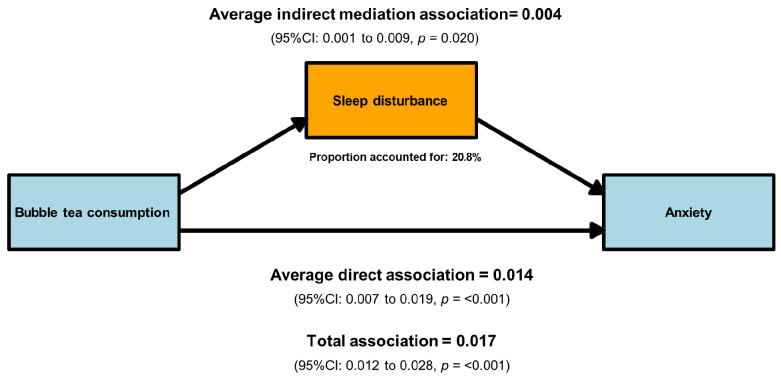
Exploratory mediation analysis of sleep disturbance in the association between bubble tea consumption and anxiety. CI = Confidence interval.

Exploratory mediation analysis was conducted to assess whether sleep disturbance statistically accounted for part of the observed association between bubble tea consumption frequency and anxiety symptoms (Figure 4). Bubble tea consumption frequency was significantly associated with anxiety symptoms (total association coefficient = 0.017, 95% CI: 0.012–0.028, *p* < 0.001). A statistically significant indirect association involving sleep disturbance was observed (coefficient = 0.004, 95% CI: 0.001–0.009, *p* < 0.05), while the association between bubble tea consumption frequency and anxiety symptoms remained significant after accounting for sleep disturbance (coefficient = 0.014, 95% CI: 0.007–0.019, *p* < 0.001). The indirect association involving sleep disturbance corresponded to approximately 20.8% (95% CI: 7.1–52.2%) of the observed association between bubble tea consumption frequency and anxiety symptoms. Accordingly, these findings should be interpreted as an exploratory statistical decomposition of cross-sectional associations rather than evidence of causal mediation, as the temporal ordering of bubble tea consumption, sleep disturbance, and anxiety symptoms could not be established.

## 4. Discussion

In this large cross-sectional study of 11,847 adolescents, approximately one-third of participants (32.03%) reported anxiety symptoms, as defined by a GAD-7 score ≥ 5. This prevalence is consistent with a previous estimate of 31.3% reported among adolescents in Zhejiang Province [8], suggesting that anxiety symptoms are highly prevalent in this population. It is important to note that the GAD-7 cut-off used in the present study identifies the presence of anxiety symptoms, including mild symptoms, rather than clinically diagnosed anxiety disorders. Consequently, the reported prevalence should not be interpreted as the prevalence of generalized anxiety disorder or other clinically confirmed anxiety conditions. Nevertheless, the findings indicate that a substantial proportion of adolescents experience elevated anxiety symptomatology, underscoring the importance of identifying behavioral and lifestyle factors that may be associated with mental health during this critical developmental period.

The present study found that higher bubble tea consumption frequency was associated with greater odds of anxiety symptoms, with evidence of a positive linear dose–response relationship. In the fully adjusted model, each additional day of bubble tea consumption per week was associated with a 10% increase in the odds of anxiety symptoms, and adolescents consuming bubble tea 3–7 days per week had 53% higher odds compared with non-consumers. These associations remained statistically significant after adjustment for sociodemographic, behavioral, and lifestyle factors. RCS analyses further demonstrated a linear increase in the odds of anxiety symptoms with increasing bubble tea consumption frequency, without evidence of nonlinearity. Sleep disturbance was strongly associated with anxiety symptoms, and exploratory analyses identified a statistically significant indirect association involving sleep disturbance corresponding to approximately 20.8% of the observed association between bubble tea consumption and anxiety symptoms. It is important to emphasize that these mediation analyses are cross-sectional and purely statistical; they do not provide evidence of causal pathways. Taken together, these findings suggest that frequent bubble tea consumption is associated with higher anxiety symptoms among adolescents, warranting further investigation in longitudinal studies with repeated assessments of dietary intake, sleep health, and psychological outcomes.

Our findings are consistent with previous studies reporting associations between bubble tea consumption and adverse mental health outcomes. For example, a multicenter cross-sectional study of 15,440 university students from four provinces in China found that bubble tea consumption was associated with higher odds of anxiety symptoms [28]. Similarly, a study of 5281 college students in Beijing reported associations between bubble tea consumption and depression, anxiety, suicidal ideation, and addictive behaviors [15]. An animal study also reported anxiety-like behaviors among mice exposed to long-term bubble tea intake [14]. Furthermore, previous studies have reported associations between bubble tea consumption and sleep-related outcomes. A multicenter study of university students from Shanghai, Jiangxi, Hubei, and Shanxi Provinces found that bubble tea consumers were more likely to report insomnia symptoms than non-consumers [29]. Consistent with these findings, higher caffeine intake has been associated with longer sleep-onset latency, reduced total sleep time, lower sleep efficiency, and reduced rapid eye movement sleep among adolescents [30]. Our findings extend the existing literature by demonstrating a graded association between bubble tea consumption frequency and anxiety symptoms, with progressively higher odds of anxiety observed at higher levels of consumption.

The strong association between sleep disturbance and anxiety symptoms observed in this study is consistent with a substantial body of literature [31]. Previous studies have shown that insomnia and other sleep problems frequently co-occur with emotional disorders in adolescents. More than half of adolescents with insomnia meet diagnostic criteria for depression or anxiety disorders [32]. Difficulty initiating sleep has been associated with attentional deficits, social withdrawal, and internalizing symptoms [33]. Short sleep duration and poor sleep quality have also been linked to higher levels of anxiety symptoms among adolescents [34]. In addition, late bedtimes have been associated with anxiety symptoms in younger populations [35]. Our findings further support the close relationship between sleep disturbance and anxiety symptoms in adolescents.

Several biological and psychosocial explanations proposed in previous research may help contextualize the associations observed in this study. Caffeine has been shown to interfere with sleep regulation through antagonism of adenosine receptors, potentially delaying sleep onset and reducing sleep duration [19,36,37]. High sugar intake has also been associated with sleep disruption through effects on metabolic regulation and blood glucose fluctuations [38,39]. In addition, diets high in added sugars have been linked to low-grade systemic inflammation, which may be associated with both sleep and emotional functioning [40,41].

Sleep disturbance has been associated with several neurobiological processes relevant to emotional regulation. Experimental and observational studies have reported that insufficient or fragmented sleep is associated with increased amygdala reactivity to negative stimuli and reduced functional connectivity between the amygdala and prefrontal cortex [42,43,44]. Chronic sleep problems have also been associated with dysregulation of the hypothalamic–pituitary–adrenal axis and elevated cortisol levels [45,46]. Although these findings provide potential explanations for the observed associations, the present cross-sectional study cannot establish temporal ordering or causal mechanisms.

Behavioral and psychosocial factors may also contribute to the observed associations. Adolescents who frequently consume bubble tea may exhibit broader lifestyle patterns characterized by irregular daily routines, greater screen exposure, and preference for energy-dense foods [47,48]. In addition, frequent consumption of sweetened beverages has been associated with reward-seeking tendencies and habitual consumption patterns [49,50]. These interconnected behavioral and psychosocial characteristics may be relevant to understanding the observed associations and warrant further investigation.

The exploratory analysis indicated a statistically significant indirect association involving sleep disturbance, which corresponded to approximately 20.8% of the observed association between bubble tea consumption frequency and anxiety symptoms. However, these findings should be interpreted with caution. Because all variables were measured at the same time point, the temporal ordering among bubble tea consumption, sleep disturbance, and anxiety symptoms could not be established. Accordingly, these results should be viewed as a statistical decomposition of cross-sectional associations rather than evidence of a mediating mechanism or causal pathway. Alternative explanations, including reverse causation, bidirectional relationships, and residual confounding, cannot be excluded.

From a public health perspective, the findings suggest that bubble tea consumption and sleep health may be relevant factors to consider in future research on adolescent mental health. Given the high prevalence of bubble tea consumption among adolescents, further investigation of its relationship with sleep and emotional well-being is warranted. However, bubble tea consumption in the present study was assessed only by consumption frequency, without detailed information on serving size, sugar content, caffeine content, tea type, toppings, or timing of consumption. Therefore, the specific components or consumption patterns underlying the observed associations remain unclear. The findings should not be interpreted as evidence that all forms of bubble tea consumption have similar relationships with sleep disturbance and anxiety symptoms. Sleep disturbance remains an important correlate of anxiety symptoms and may represent a promising target for future prevention and intervention efforts. Nevertheless, whether reducing bubble tea consumption, modifying specific beverage characteristics, or improving sleep quality can lead to improvements in anxiety symptoms cannot be determined from the present cross-sectional study and requires confirmation in longitudinal and intervention studies.

This study has several strengths. The large school-based sample provided substantial statistical power to evaluate associations across multiple exposure categories and conduct subgroup analyses. The study incorporated adjustment for a broad range of sociodemographic and lifestyle factors, including screen time, fast-food consumption, and academic performance. In addition, the use of the validated GAD-7 instrument, RCS modeling, and exploratory mediation analysis allowed for a more comprehensive examination of the observed associations.

However, several limitations should be considered. First, the cross-sectional design precludes causal inference, and reverse causation (e.g., anxiety leading to increased bubble tea consumption or sleep disturbance) cannot be ruled out. Second, sleep disturbance and other study variables were self-reported, which may have introduced recall and reporting bias. In addition, sleep disturbance was assessed using a brief symptom-based questionnaire rather than validated sleep instruments or objective measures such as actigraphy or polysomnography. Because sleep disturbance was a key variable in the analyses, including the examination of its potential role in the association between bubble tea consumption and anxiety, measurement error or misclassification may have influenced the observed findings. Therefore, the results involving sleep disturbance should be interpreted with caution and warrant confirmation using validated sleep assessments and objective sleep measures. Third, despite adjustment for multiple covariates, residual confounding remains possible. Unmeasured factors, including overall dietary patterns, total caffeine intake, chronotype, peer influences, family stress, depressive symptoms, and genetic susceptibility, may partly explain the observed associations. Fourth, bubble tea consumption was measured only by frequency (number of days per week), and the questionnaire did not include information on serving size, caffeine content, sugar content, tea type, toppings, or timing of consumption. More detailed assessment of these characteristics would improve exposure characterization and help clarify which components may be most relevant to mental health outcomes. Finally, the primary outcome was defined using a GAD-7 score ≥ 5, capturing anxiety symptoms, including mild cases, rather than clinically diagnosed anxiety disorders. Consequently, the prevalence of clinically significant anxiety may have been overestimated, and this distinction should be considered when interpreting the findings.

Future research should seek to replicate these findings using longitudinal and prospective study designs to clarify temporal relationships. The incorporation of validated sleep instruments, objective sleep assessments such as actigraphy, and more detailed measures of dietary intake, including biomarkers of caffeine or sugar exposure, would improve measurement precision. In addition, intervention studies are needed to determine whether modifying bubble tea consumption or improving sleep quality influences anxiety symptoms among adolescents.

## 5. Conclusions

In conclusion, this cross-sectional study found that higher bubble tea consumption frequency was associated with greater odds of anxiety symptoms among adolescents in a positive linear dose–response pattern. Exploratory analyses identified a statistically significant indirect association involving sleep disturbance. These findings should be interpreted as exploratory and hypothesis-generating. Further longitudinal and prospective studies are needed to clarify the temporal sequence and potential mechanisms underlying these relationships.

## Figures and Tables

**Table 1:** Basic characteristics of the students stratified by anxiety status (*n* = 11,847).

Characteristics	Total (*n* = 11,847)	Students Without Anxiety (*n* = 8052)	Students with Anxiety (*n* = 3795)	t/χ^2^	*p*-Value
Age (years), [means ± SD]	13.66 ± 1.49	13.51 ± 1.42	13.98 ± 1.60	−15.49 ^a^	<0.001 ***
Sex, *n* (%)				166.68 ^b^	<0.001 ***
Boy	6365 (53.73)	4653 (57.79)	1712 (45.11)		
Girl	5482 (46.27)	3399 (42.21)	2083 (54.89)		
BMI category, *n* (%)				4.93 ^b^	0.026 *
Normal	8831 (74.54)	5953 (73.93)	2878 (75.84)		
Overweight/obesity	3016 (25.46)	2099 (26.07)	917 (24.16)		
Residence, *n* (%)				1.32 ^b^	0.251
Rural	7702 (65.01)	5207 (64.67)	2495 (65.74)		
Urban	4145 (34.99)	2845 (35.33)	1300 (34.26)		
School type, *n* (%)				191.48 ^b^	<0.001 ***
Middle school	9609 (81.11)	6806 (84.53)	2803 (73.86)		
High school	2238 (18.89)	1246 (15.47)	992 (26.14)		
Only Child, *n* (%)				2.56 ^b^	0.110
No	8360 (70.57)	5645 (70.11)	2715 (71.54)		
Yes	3487 (29.43)	2407 (29.89)	1080 (28.46)		
Accommodation, *n* (%)				178.10 ^b^	<0.001 ***
Others	8005 (67.57)	5758 (71.51)	2247 (59.21)		
School dormitory	3842 (32.43)	2294 (28.49)	1548 (40.79)		
Father’s education level, *n* (%)				73.20 ^b^	<0.001 ***
Secondary school and lower	4299 (36.29)	2760 (34.28)	1539 (40.55)		
Senior high school	3575 (30.18)	2397 (29.77)	1178 (31.04)		
College or above	3973 (33.53)	2895 (35.95)	1078 (28.41)		
Mother’s education level, *n* (%)				91.56 ^b^	<0.001 ***
Secondary school and lower	4639 (39.16)	2930 (36.39)	1709 (45.03)		
Senior high school	3168 (26.74)	2187 (27.16)	981 (25.85)		
College or above	4040 (34.10)	2935 (36.45)	1105 (29.12)		
Bubble tea consumption frequency (days/week), *n* (%)				89.87 ^b^	<0.001 ***
0	5152 (43.49)	3682 (45.72)	1470 (38.74)		
1–2	5822 (49.14)	3880 (48.19)	1942 (51.17)		
≥3	873 (7.37)	490 (6.09)	383 (10.09)		
Sleep disturbance, *n* (%)	3319 (28.02)	1309 (16.26)	2010 (52.96)	1723.38 ^b^	<0.001 ***
Smoking, *n* (%)	197 (1.66)	76 (0.94)	121 (3.19)	79.47 ^b^	<0.001 ***
Drinking, *n* (%)	1262 (10.65)	657 (8.16)	605 (15.94)	164.14 ^b^	<0.001 ***
Regular exercise, *n* (%)	6661 (56.23)	4744 (58.92)	1917 (50.51)	74.00 ^b^	<0.001 ***
Fast-food consumption, *n* (%)	5684 (47.98)	3730 (46.32)	1954 (51.49)	27.57 ^b^	<0.001 ***
Screen time (hours), *n* (%)				83.10 ^b^	<0.001 ***
<2	8283 (69.92)	5842 (72.55)	2441 (64.32)		
≥2	3564 (30.08)	2210 (27.45)	1354 (35.68)		
Self-rated academic performance, *n* (%)				163.20 ^b^	<0.001 ***
Excellent	2466 (20.82)	1841 (22.86)	625 (16.47)		
Average	5979 (50.47)	4175 (51.85)	1804 (47.54)		
Below average	3402 (28.71)	2036 (25.29)	1366 (35.99)		

* *p* < 0.05; *** *p* < 0.001. ^a^ Student’s *t*-test; ^b^ Chi-square test. Abbreviations: BMI, body mass index; SD, standard deviation.

**Table 2:** Prevalence of anxiety, bubble tea consumption and sleep disturbance by participant characteristics (*n* = 11,847).

Characteristics	N (%)	Anxiety, *n* (%)	*p*-Value	Bubble Tea Consumption (≥1 Day/Week), *n* (%)	*p*-Value	Sleep Disturbance, *n* (%)	*p*-Value
Age group (years)			<0.001 ***		0.828		<0.001 ***
≤13	6605 (55.75)	1766 (26.74)		3731 (56.49)		1634 (24.74)	
14–15	3490 (29.46)	1224 (35.07)		1963 (56.25)		1034 (29.63)	
≥16	1752 (14.79)	805 (45.95)		1001 (57.13)		651 (37.16)	
Sex			<0.001 ***		<0.001 ***		<0.001 ***
Boy	6365 (53.73)	1712 (26.90)		3275 (51.45)		1665 (26.16)	
Girl	5482 (46.27)	2083 (38.00)		3420 (62.39)		1654 (30.17)	
Residence			0.251		<0.001 ***		0.298
Rural	7702 (65.01)	2495 (32.39)		4118 (53.47)		2182 (28.33)	
Urban	4145 (34.99)	1300 (31.36)		2577 (62.17)		1137 (27.43)	
BMI category			0.026 *		0.485		0.001 **
Normal	8831 (74.54)	2878 (32.59)		5007 (56.70)		2404 (27.22)	
Overweight/obesity	3016 (25.46)	917 (30.40)		1688 (55.97)		915 (30.34)	
Screen time			<0.001 ***		<0.001 ***		<0.001 ***
<2	8283 (69.92)	2441 (29.47)		4464 (53.89)		2004 (24.19)	
≥2	3564 (30.08)	1354 (37.99)		2231 (62.60)		1315 (36.90)	
Smoking			<0.001 ***		<0.001 ***		<0.001 ***
No	11650 (98.34)	3674 (31.54)		6561 (56.32)		3198 (27.45)	
Yes	197 (1.66)	121 (61.42)		134 (68.02)		121 (61.42)	
Drinking			<0.001 ***		<0.001 ***		<0.001 ***
No	10585 (89.35)	3190 (30.14)		5861 (55.37)		2775 (26.22)	
Yes	1262 (10.65)	605 (47.94)		834 (66.09)		544 (43.11)	
Fast-food consumption			<0.001 ***		<0.001 ***		0.068
No	6163 (52.02)	1841 (29.87)		2568 (41.67)		1682 (27.29)	
Yes	5684 (47.98)	1954 (34.38)		4127 (72.61)		1637 (28.80)	
Regular exercise			<0.001 ***		0.069		<0.001 ***
No	5186 (43.77)	1878 (36.21)		2882 (55.57)		1689 (32.57)	
Yes	6661 (56.23)	1917 (28.78)		3813 (57.24)		1630 (24.47)	
Self-rated academic performance			<0.001 ***		<0.001 ***		<0.001 ***
Excellent	2466 (20.82)	625 (25.34)		1337 (54.22)		522 (21.17)	
Average	5979 (50.47)	1804 (30.17)		3347 (55.98)		1472 (24.62)	
Below average	3402 (28.71)	1366 (40.15)		2011 (59.11)		1325 (38.95)	

* *p* < 0.05; ** *p* < 0.01; *** *p* < 0.001. Abbreviations: BMI, body mass index.

**Table 3:** Multivariable logistic regression analysis of factors associated with anxiety (*n* = 11,847).

Characteristics	Total (*n*)	Anxiety (*n*)	OR	95% CI	*p*-Value
Age group (years)					
≤13	6605	1766	1.00 (Reference)	1.00 (Reference)	
14–15	3490	1224	1.41	1.27–1.57	<0.001 ***
≥16	1752	805	2.09	1.63–2.68	<0.001 ***
Sex					
Boy	6365	1712	1.00 (Reference)	1.00 (Reference)	
Girl	5482	2083	1.67	1.52–1.82	<0.001 ***
BMI category					
Normal	8831	2878	1.00 (Reference)	1.00 (Reference)	
Overweight/obesity	3016	917	0.89	0.81–0.99	0.029 *
School type					
Middle school	9609	2803	1.00 (Reference)	1.00 (Reference)	
High school	2238	992	0.87	0.70–1.09	0.215
Residence					
Rural	7702	2495	1.00 (Reference)	1.00 (Reference)	
Urban	4145	1300	0.91	0.82–1.01	0.078
Only Child					
No	8360	2715	1.00 (Reference)	1.00 (Reference)	
Yes	3487	1080	1.04	0.95–1.15	0.410
Accommodation, *n* (%)					
Others	8005	2247	1.00 (Reference)	1.00 (Reference)	
School dormitory	3842	1548	1.25	1.13–1.39	<0.001 ***
Father’s education level					
Secondary school and lower	4299	1539	1.00 (Reference)	1.00 (Reference)	
Senior high school	3575	1178	1.03	0.92–1.16	0.596
College or above	3973	1078	0.92	0.80–1.06	0.238
Mother’s education level					
Secondary school and lower	4639	1709	1.00 (Reference)	1.00 (Reference)	
Senior high school	3168	981	0.83	0.73–0.93	0.001 **
College or above	4040	1105	0.83	0.73–0.95	0.008 **
Bubble tea consumption (days/week)					
0	5152	1470	1.00 (Reference)	1.00 (Reference)	
1–2	5822	1942	1.11	1.01–1.22	0.029 *
≥3	873	383	1.41	1.19–1.68	<0.001 ***
Sleep disturbance					
No	8528	1785	1.00 (Reference)	1.00 (Reference)	
Yes	3319	2010	5.00	4.57–5.48	<0.001 ***
Smoking					
No	11,650	3674	1.00 (Reference)	1.00 (Reference)	
Yes	197	121	1.73	1.24–2.40	0.001 **
Drinking					
No	10,585	3190	1.00 (Reference)	1.00 (Reference)	
Yes	1262	605	1.57	1.37–1.80	<0.001 ***
Screen time (hours)					
<2	8283	2441	1.00 (Reference)	1.00 (Reference)	
≥2	3564	1354	1.09	0.99–1.20	0.091
Self-rated academic performance					
Excellent	2466	625	1.00 (Reference)	1.00 (Reference)	
Average	5979	1804	1.17	1.04–1.31	0.010 **
Below average	3402	1366	1.37	1.21–1.56	<0.001 ***
Regular exercise					
No	5186	1878	1.00 (Reference)	1.00 (Reference)	
Yes	6661	1917	0.86	0.78–0.93	<0.001 ***
Fast-food consumption					
No	6163	1841	1.00 (Reference)	1.00 (Reference)	
Yes	5684	1954	1.15	1.05–1.26	0.003 **

* *p* < 0.05; ** *p* < 0.01; *** *p* < 0.001. Abbreviations: OR, odds ratio; CI, confidence interval; BMI, body mass index.

**Table 4:** Multivariable regression analysis of bubble tea consumption frequency in relation to anxiety (*n* = 11,847).

Characteristics	Model 1		Model 2		Model 3	
	OR (95% CI)	*p*-Value	OR (95% CI)	*p*-Value	OR (95% CI)	*p*-Value
Bubble tea consumption frequency (days/week) (continuous)	1.17 (1.13–1.21)	<0.001 ***	1.16 (1.12–1.20)	<0.001 ***	1.10 (1.06–1.14)	<0.001 ***
Bubble tea consumption frequency (days/week) (category)						
0	1.00 (Reference)		1.00 (Reference)		1.00 (Reference)	
1–2	1.25 (1.16–1.36)	<0.001 ***	1.18 (1.09–1.28)	<0.001 ***	1.12 (1.02–1.22)	0.014 *
≥3	1.96 (1.69–2.27)	<0.001 ***	1.93 (1.66–2.24)	<0.001 ***	1.53 (1.30–1.80)	<0.001 ***
*p* for trend		<0.001 ***		<0.001 ***		<0.001 ***

* *p* < 0.05; *** *p* < 0.001. Abbreviations: OR, odds ratio; CI, confidence interval. Model 1: unadjusted analysis (no covariates included); Model 2: adjusted for age and sex; Model 3: adjusted for age, sex, BMI, residence, parental education, school type, dormitory status, only-child status, smoking, drinking, fast-food consumption, self-rated academic performance, screen time, and regular exercise.

## Data Availability

The data presented in this study are available on request from the corresponding author (the data are not publicly available due to privacy restrictions).

## Supplementary Materials

The following supporting information can be downloaded at https://www.mdpi.com/article/10.3390/nu18121960/s1, Table S1: Multilevel logistic regression analysis of factors associated with anxiety (random intercepts for schools) (*n* = 11,847); Table S2: Multivariable logistic regression analysis of bubble tea consumption frequency in relation to moderate-to-severe anxiety (GAD-7 ≥ 10) (*n* = 11,847); Figure S1: Dose–response association between weekly bubble tea consumption days and moderate-to-severe anxiety (GAD-7 ≥ 10).

## Abbreviations

The following abbreviations are used in this manuscript:

GAD-7	Generalized Anxiety Disorder 7
RCS	Restricted cubic spline
OR	Odds ratio
CI	Confidence interval
BMI	Body mass index
ANOVA	Analysis of variance
SD	Standard deviation
