# Supplementary material for: Associations of Bubble Tea Consumption with Sleep Disturbance and Anxiety in Adolescents: Findings from the Zhejiang Childhood Behavior and Health Cohort
Source: Nutrients. 2026 Jun 17;18(12):1960. doi: 10.3390/nu18121960 (PMC13304769; doi:10.3390/nu18121960)
Supplement: Supplementary file 1 [file nutrients-18-01960-s001.zip › Supplementary File.pdf]

Supplementary:

Table S1. Multilevel logistic regression analysis of factors associated with anxiety (random intercepts for schools)(n=11,847)

Characteristics	Total (n)	Anxiety (n)	OR	95%CI	*p*-value
Age group (years)					
≤13	6,605	1,766	1.00(Reference)	1.00(Reference)	
14-15	3,490	1,224	1.39	1.25-1.55	<0.001***
≥16	1,752	805	2.41	1.87-3.11	<0.001***
Sex					
Boy	6,365	1,712	1.00(Reference)	1.00(Reference)	
Girl	5,482	2,083	1.67	1.53-1.82	<0.001***
BMI category					
Normal	8,831	2,878	1.00(Reference)	1.00(Reference)	
Overweight/obesity	3,016	917	0.88	0.80-0.98	0.014*
School type					
Middle school	9,609	2,803	1.00(Reference)	1.00(Reference)	
High school	2,238	992	0.89	0.60-1.32	0.555
Residence					
Rural	7,702	2,495	1.00(Reference)	1.00(Reference)	
Urban	4,145	1,300	0.90	0.65-1.25	0.523
Only Child					
No	8,360	2,715	1.00(Reference)	1.00(Reference)	
Yes	3,487	1,080	1.03	0.93-1.14	0.553
Accommodation,*n*(%)					
Others	8,005	2,247	1.00(Reference)	1.00(Reference)	
School dormitory	3,842	1,548	1.03	0.91-1.17	0.642
Father's education level					
Secondary school and lower	4,299	1,539	1.00(Reference)	1.00(Reference)	
Senior high school	3,575	1,178	1.07	0.95-1.20	0.293

College or above	3,973	1,078	0.99	0.86-1.14	0.890
Mother's education level					
Secondary school and lower	4,639	1,709	1.00(Reference)	1.00(Reference)	
Senior high school	3,168	981	0.85	0.76-0.96	0.009**
College or above	4,040	1,105	0.90	0.79-1.04	0.149
Bubble tea consumption (days/week)					
0	5,152	1,470	1.00(Reference)	1.00(Reference)	
1-2	5,822	1,942	1.15	1.05-1.26	0.003**
≥3	873	383	1.45	1.23-1.72	<0.001***
Sleep disturbance					
No	8,528	1,785	1.00(Reference)	1.00(Reference)	
Yes	3,319	2,010	5.00	4.56-5.48	<0.001***
Smoking					
No	11,650	3,674	1.00(Reference)	1.00(Reference)	
Yes	197	121	1.55	1.11-2.17	0.010*
Drinking					
No	10,585	3,190	1.00(Reference)	1.00(Reference)	
Yes	1,262	605	1.53	1.33-1.76	<0.001***
Screen time (hours)					
<2	8,283	2,441	1.00(Reference)	1.00(Reference)	
≥2	3,564	1,354	1.06	0.96-1.17	0.268
Self-rated academic performance					
Excellent	2,466	625	1.00(Reference)	1.00(Reference)	
Average	5,979	1,804	1.19	1.06-1.34	0.004**
Below average	3,402	1,366	1.42	1.25-1.62	<0.001***
Regular exercise					

No	5,186	1,878	1.00(Reference)	1.00(Reference)	
Yes	6,661	1,917	0.86	0.79-0.94	<0.001***
Fast-food consumption					
No	6,163	1,841	1.00(Reference)	1.00(Reference)	
Yes	5,684	1,954	1.19	1.08-1.30	<0.001***

*p<0.05; **p<0.01; ***p<0.001.

Abbreviations: OR, odds ratio; CI, confidence interval; ref, reference; BMI, body mass index.

Table S2. Multivariable regression analysis of bubble tea consumption frequency in relation to moderate-to-severe anxiety (GAD-7 ≥10) (n=11,847)

Characteristics	Model 1		Model 2		Model 3	
	OR(95%CI)	*p*-value	OR(95%CI)	*p*-value	OR(95%CI)	*p*-value
Bubble tea consumption frequency (days/week)(continuous)	1.19(1.13-1.24)	<0.001***	1.18(1.12-1.23)	<0.001***	1.09(1.03-1.15)	0.002**
Bubble tea consumption frequency(days/week)(category)						
0	1.00(Reference)		1.00(Reference)		1.00(Reference)	
1-2	1.15(1.01-1.32)	0.035*	1.07(0.94-1.22)	0.323	0.99(0.86-1.15)	0.932
≥3	2.12(1.72-2.60)	<0.001***	2.03(1.65-2.50)	<0.001***	1.46(1.16-1.83)	0.001**
P for trend		<0.001***		<0.001***		0.003**

*p<0.05; **p<0.01; ***p<0.001.

Abbreviations: OR, odds ratio; CI, confidence interval; ref, reference.

Model 1: unadjusted analysis (no covariates included); Model 2: adjusted for age, sex; Model 3: adjusted for age, sex, BMI, residence, parental education, school type, dormitory status, only-child status, smoking, drinking, fast-food consumption, self-rated academic performance, screen time, and regular exercise.

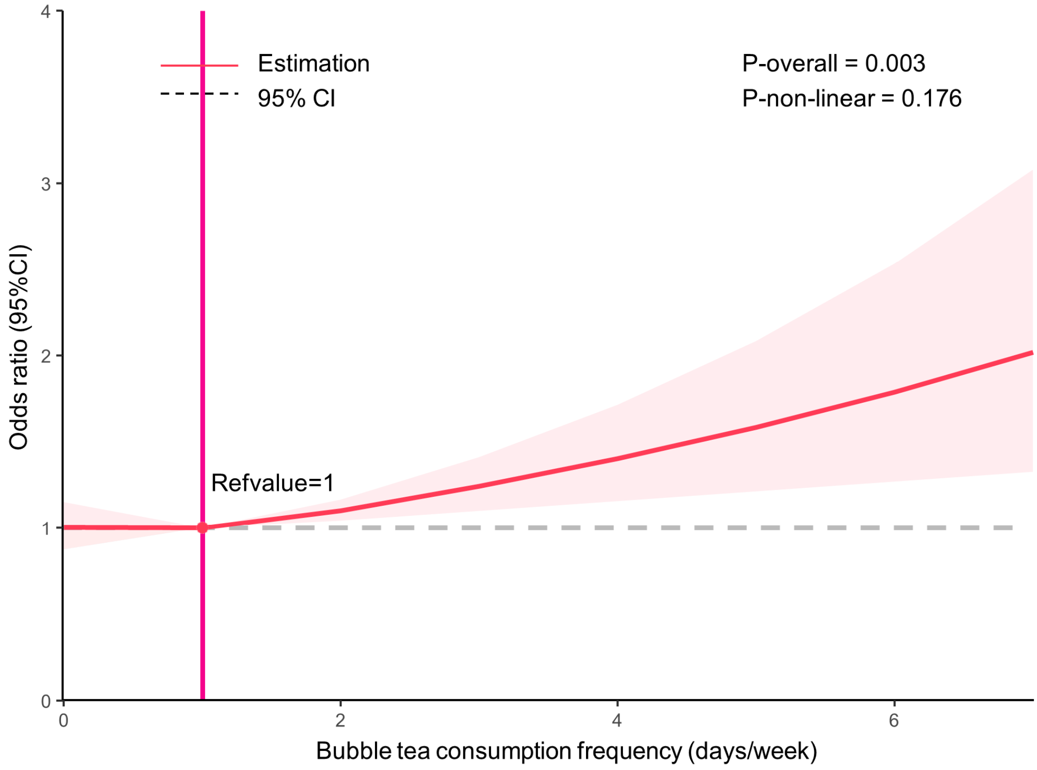

Figure S1.Dose-response association between weekly bubble tea consumption days and moderate-to-severe anxiety (GAD-7 ≥10). Restricted cubic spline (RCS) model was used to estimate the association between weekly bubble tea consumption days (0–7 days/week) and the odds of anxiety, with 95% confidence intervals (CIs). The x-axis represents weekly bubble tea consumption days, and the y-axis represents the odds ratios (ORs) for anxiety. The solid line indicates the estimated association, and the shaded area represents the corresponding 95% CIs. The horizontal dashed line at OR = 1.0 indicates no association. The vertical solid line indicates the 50th percentile (median; reference value) of weekly bubble tea consumption days (1 day/week). Model was adjusted for age, sex, BMI, residence, parental education, school type, dormitory status, only-child status, smoking, drinking, fast-food consumption, self-rated academic performance, screen time, and regular exercise.
